# Two analytical approaches for determination of amprolium and triclabendazole targeting their tertiary amino groups in waste water

**DOI:** 10.1038/s41598-025-22052-9

**Published:** 2025-10-28

**Authors:** Maram Attia, Randa A. Abdel Salam, Ghada M. Hadad, Mary E. K. Wahba

**Affiliations:** 1 Pharmaceutical Analytical Chemistry Department, Faculty of Pharmacy, Suez Canal University, Ismailia, Egypt; 2 Pharmaceutical Chemistry Department, Faculty of Pharmacy, Delta University for Science and Technology, Gamasa, Egypt

**Keywords:** Amprolium, Triclabendazole, Eosin-Y, Spectrophotometry, Spectrofluorimetry, Wastewater, Chemistry, Environmental sciences

## Abstract

This study has been proposed new, sensitive, straightforward, and validated spectrophotometric and spectrofluorimetric methods for estimating of amprolium (AMP) and triclabendazole (TCB) in laboratory prepared pharmaceutical wastewater. Both methods were based on the reaction of AMP and TCB with Eosin-Y in acidic medium, targeting the tertiary amino groups of the concerned analytes, generating an orange-red ion pair complexes. The resulting products were measured by spectrophotometric (Method I) and spectrofluorimetric (Method II) tools. Regarding method I, the absorbance was measured at 547 nm with a lower detection limit of 45 ng/mL and quantitation limit of 136 ng/mL for AMP with corresponding values of 191 ng/mL and 579 ng/mL for TCB. The proposed method exhibited a linear pattern over the concentration range of 100–5000 and 1500–10,000 ng/mL for amprolium and triclabendazole, respectively. Meanwhile in method II, the produced complex was measured at λem 552 nm for AMP or 555 nm for TCB after excitation at λex 470 nm with a lower detection limit of 142 ng/mL and quantitation limit of 429 ng/mL for AMP with corresponding values of 67 ng/mL and 202 ng/mL for TCB. The proposed method exhibited a linear pattern over the concentration range with linearity ranges of 500–2000 and 100–1500 ng/mL for amprolium and triclabendazole, respectively. The International Conference on Harmonization (ICH) requirements for linearity, accuracy, precision, specificity, and robustness were followed in the comprehensive validation of the proposed methods, allowing its application successfully to determine the studied analytes in wastewater samples.

## Introduction

**Fig. 1:**
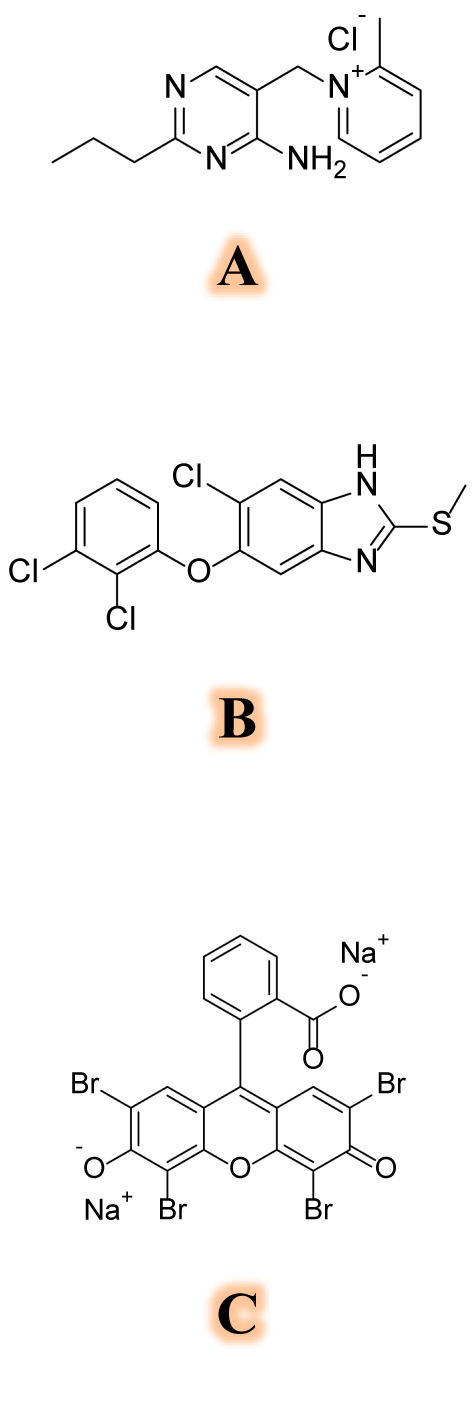
Structural formulae of AMP (**A**), TCB (**B**) and Eosin Y (**C**).

Amprolium (AMP); 5-[(2-methylpyridin-1-ium-1-yl) methyl]-2-propylpyrimidin-4 amine;chloride; hydrochloride^1^ (Fig. 1A) belongs to methylpyridine class of organic compounds. At lower doses, it has a coccidiostatic effect; while at higher doses, it exhibits a coccidiocidal effect^2^. As a thiamine antagonist, this quaternized derivative of pyrimidine inhibits thiamine metabolism by obstructing thiamine receptors, thus hinders the synthesis of carbohydrates. It is a control measure for coccidiosis in chicken feed^3^.

Triclabendazole (TCB); 5-Chloro-6-(2,3-dichlorophenoxy)-2-(methylthio)-1H-benzimidazole^1^ (Fig. 1B) belongs to the class of anthelmintics. It is recommended for the management of paragonimiasis and fascioliasis^4^.

Determination of AMP and TCB, whether alone or in combination with other drugs, was previously dwelled applying various approaches. Some selected examples of these methods include: spectrophotometry^5–9^, high performance liquid chromatography HPLC^10–21^, high performance thin layer chromatography^22–24^ and liquid chromatography-tandem mass spectrometry^25–30^.

Despite its many benefits, liquid chromatography needs skilled analysts to operate, constant maintenance, uses costly, high-grade chemicals, and consumes a lot of organic solvents which deprive it from the required sustainability. On the other hand, spectrophotometry is a popular analytical instrument because of its affordability, accessibility in most laboratories, ease of use, and simplicity. These facts encouraged the authors to use colorimetry (Method I) for the assay of AMP and TCB; where the suggested approach resulted in a wide linearity range, showed higher sensitivity measures than the reported assays, as indicated by the resultant LOD and LOQ values.

Furthermore, an alternative to these methods is still required for the routine quality control analysis of the drugs in concern. Because of their ease of use, low equipment costs, great sensitivity, and broad working concentration range, spectrofluorimetric techniques have received more interest which could be manifested from previously published articles^31–34^. These factors led us to carry out the current investigation (Method II).

Eosin Y (Fig. 1C) is a xanthene dye, which reacts with analytes containing tertiary amino groups producing ion-pair complexes^35^. It has been reported that in acidic conditions, stable complexes with cationic basic nitrogenous drugs quench the fluorescence of Eosin Y^36–42^. A good deal to the application of Eosin Y for the assay of various pharmaceutical compounds could be found in the review article presented by Derayea SM et al.^43^.

Because of the relevance of pharmaceutical wastewater application, it should receive more attention, hence it was the target for our application in this work.

## Experimental

### Instrument

- Shimadzu (Kyoto, Japan) UV-1900i PC, UV–Visible double-beam spectrophotometer.
- Shimadzu RF-6000 (A40245801833SA) Spectro fluorophotometer equipped with a 150W Xenon arc lamp, (Shimadzu, Kyoto, Japan).
- The pH was adjusted using a “Jenway 3503 digital pH meter (Stone, Staffs, UK)”.
- “Stakpure Pure water system OmniaTap12 UV” used to get deionized water.
- “Sartorius Entris 224-1S laboratory balance” used to weigh the raw materials of drugs.

### Materials and reagents

- AMP (Batch # 20,161,002) was kindly provided by (Adwia Pharmaceutical Company, Cairo, Egypt).
- TCB was kindly supplied by (Pharma Swede-Egypt, 10th of Ramadan City, Sharqia, Egypt).
- Eosin Y was freshly prepared in deionized water as (5 × 10^–3^ M) and was purchased from (Merck, Darmstadt, Germany).
- Methanol (99.9% purity, a product of Fisher scientific, UK) HPLC grade.
- Acetic Acid was kindly supplied by (ADVENT CHEMBIO PVT.LTD, Navi Mumbai, India).
- Sodium Acetate was kindly provided by (LOBA Chemie PVT. LTD, Mumbai, India).
- Ascorbic acid was kindly supplied by (Danny Dye Chem, Chennai, India).
- Citric acid was kindly provided by (LOBA Chemie PVT. LTD, Mumbai, India).
- Saccharose was supplied by (Merck, Darmstadt, Germany).
- Disodium hydrogen phosphate was kindly provided by (PIOCHEM Laboratory Chemicals, 1st industrial zone, 6th of October, Egypt).
- Sodium chloride was kindly supplied by (PIOCHEM Laboratory Chemicals, 1st industrial zone, 6th of October, Egypt).
- HCl was supplied by (Fisher scientific, UK).
- NaOH was provided by (Sigma Aldrich).

### Standard solutions

Fresh AMP and TCB standard stock solutions were prepared by dissolving 25.0 mg of the pure drug in either deionized water or methanol respectively^44^, and completing the volume to the mark with the same solvent using a volumetric flask 25.0 mL, which yielded solutions of concentrations 1.0 mg/mL (Method I). While for (Method II), serial dilution was performed with either deionized water or methanol to reach stock solutions of concentrations 100.0 μg /mL. Working standard solutions for AMP and TCB were obtained by further dilution using deionized water or methanol respectively.

### General procedures

#### Construction of calibration curves

To cover the concentration ranges mentioned in (Table 1), appropriate volumes of AMP and TCB standard stock solution were put into a set of 10.0 mL volumetric flasks. Then appropriate volumes of (5 × 10^–3^ M) Eosin Y were added (Table 1), followed by addition of suitable volumes of 2 M acetate buffer of the specified pH (Table 1). The reaction mixtures were then diluted to the appropriate volume using deionized water. For (Method Ⅰ), each solution was measured at 547 nm against a reagent blank. The responses obtained were then plotted versus the drug’s final concentration in ng/mL, and the regression equation was derived. For (Method II), After AMP and TCB were excited at λex 470 nm, the ΔF values were measured at λem 552 nm and 555 nm respectively. The calibration plot was obtained by plotting ΔF values versus the final drug concentrations in ng/mL, followed by derivation of regression equations.

**Table 1:** Experimental reaction conditions for Amprolium and Triclabendazole applying the proposed methods.

Instrument				
Parameters	Spectrophotometry (Method Ι)		Spectrofluorimetr (Method ΙΙ)	
	AMP	TCB	AMP	TCB
Concentration range (ng/mL)	100–5000	1500–10,000	500–2000	100–1500
Buffer pH	3.5	2.5	3	3
Buffer volume (mL)	1	1	3	3
Eosin Y volume (mL)	2.7	1.5	1	1

#### Application to wastewater

Wastewater from pharmaceutical companies was prepared to mimic the composition of pharmaceutical wastewater (PWW). It was prepared using tap water spiked with 30 mg ascorbic acid, 50 mg citric acid, 100 mg saccharose, 230 mg disodium hydrogen phosphate, and 1 gm sodium chloride. The pH was then adjusted to 7 with 0.1 M HCl and 0.1 M NaOH. These procedures were followed based on an early published article^45^.

The procedures outlined in the “Construction of Calibration curves” were followed, and the regression equation was used to estimate the nominal contents of AMP and TCB in PWW applying both methods.

## Results and discussion

Without requiring any previous preparation or extraction steps, it was possible to implement two straightforward methods that allow us to assay AMP and TCB in PWW with simple procedures and reasonably priced and accessible reagents. Eosin Y reagent reacts with both AMP and TCB to produce a product with a high molar absorptivity. Additionally, the same products exhibit fluorescence. The optimization of reaction conditions was performed by varying one factor while maintaining the others constant in order to reach the greatest sensitivity parameters. Eosin-Y is expected to form an ion pair complex by combining with the tertiary amino groups of AMP and TCB in acidic medium. The produced colored complex in method I was detected at 547 nm, while in method II, the produced complex resulted in quenching of the fluorescence of Eosin Y was measured at λem 552 nm or 555 nm for AMP and TCB respectively after excitation at λex 470 nm.

### Optimization of reaction conditions

Several experimental conditions that affected the reaction in both methods were investigated and optimized.

#### Effect of pH

**Fig. 2:**
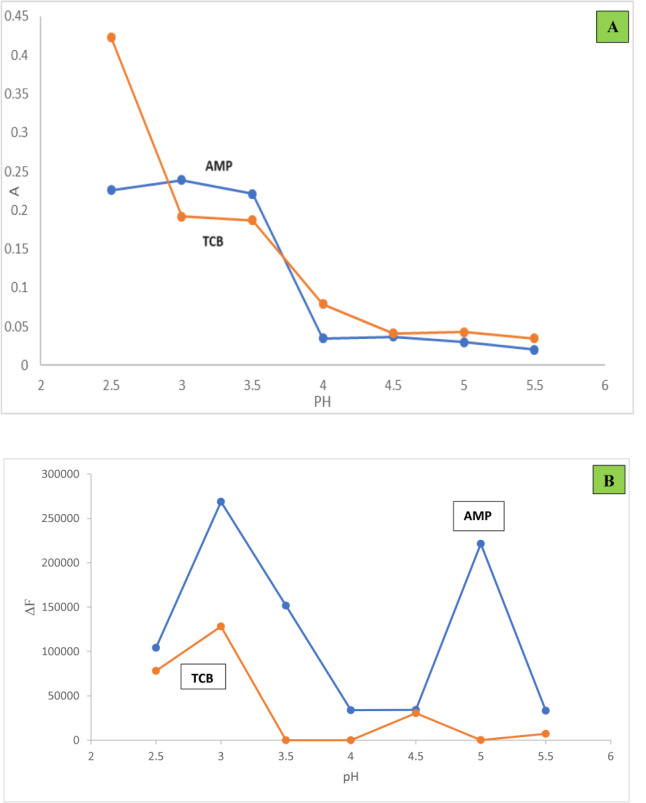
(**A**) Effect of buffer pH on the absorbance readings (method Ι) of the formed complexes (3.0 µg/mL AMP and 5.0 µg/mL TCB) using the optimum experimental conditions. (**B**) Effect of buffer pH on the ΔF values (method ΙΙ) of the formed complexes (1.0 µg/mL AMP and 0.5 µg/mL TCB) using 2.7 mL and 1.5 mL Eosin Y for AMP and TCB respectively.

The effect of pH of 2 M acetate buffer studied from 2.5 to 5.5. Regarding method I, pH 3.5 and 2.5 for AMP and TCB respectively were found to give the maximum absorbance readings. As pH is raised further, the observed responses gradually decrease (Fig. 2A), in spite that, pH 3 resulted in higher absorbance readings for AMP, it was not use; since the resultant spectrum were assymetric (broad). While for method II, maximum ΔF values were obtained at pH 3, hence it was used through this method (Fig. 2B).

#### Effect of buffer volume

**Fig. 3:**
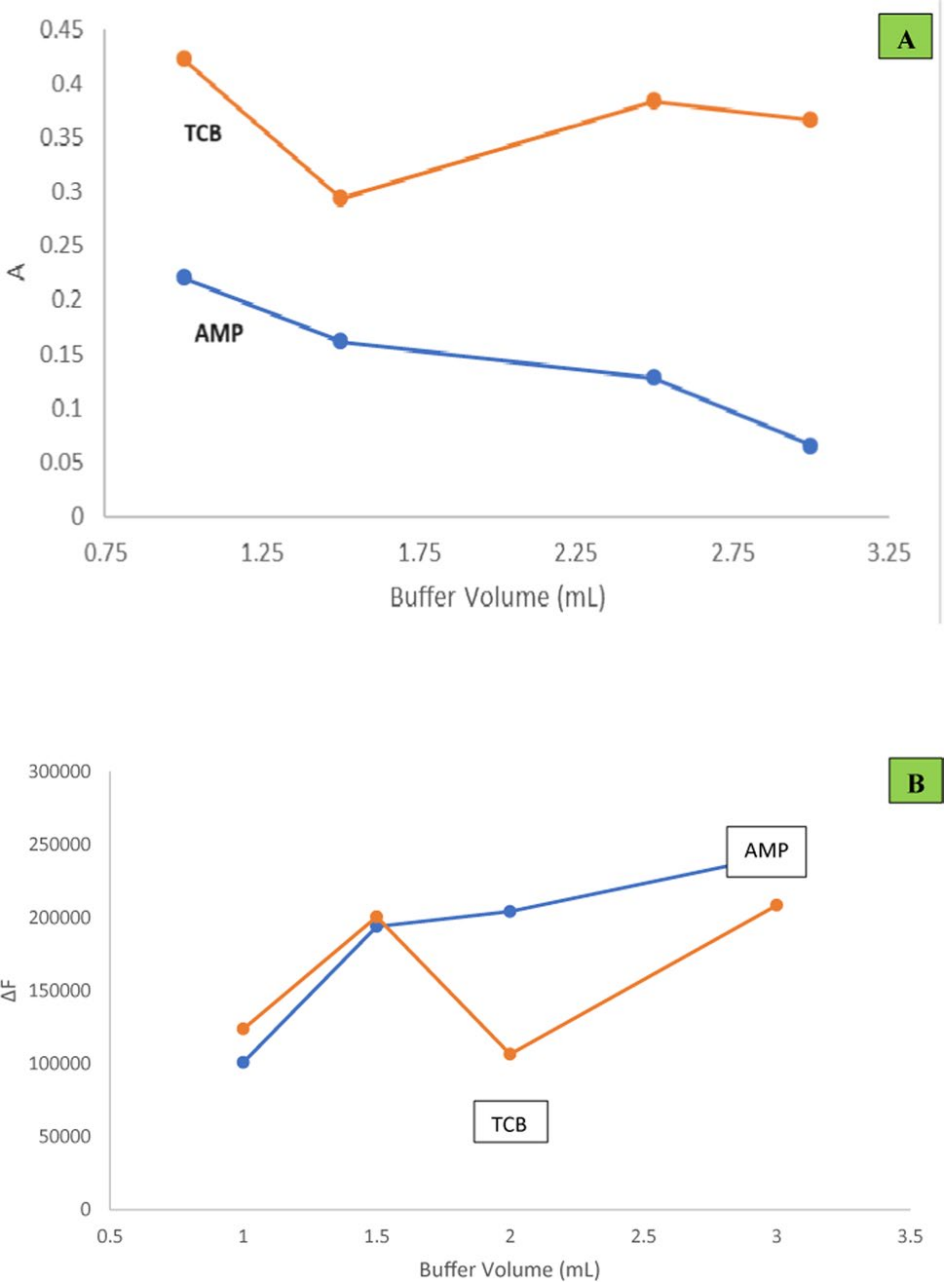
(**A**) Effect of buffer volume on the absorbance readings (method Ι) of the formed complexes (3.0 µg/mL AMP and 5.0 µg/mL TCB) using the optimum experimental conditions. (**B**) Effect of buffer volume on the ΔF values (method ΙΙ) of the formed complexes (1.0 µg/mL AMP and 0.5 µg/mL TCB) using 2.7 mL and 1.5 mL Eosin Y for AMP and TCB respectively.

The effect of volume of 2 M acetate buffer was also investigated using 1–3 mL of the buffer. Regarding method I, 1 mL (pH 3.5 for AMP and pH 2.5 for TCB) was chosen producing the highest absorbance values for the resultant complex (Fig. 3A), while for method II, the effect of the volume of acetate buffer (pH 3) on the fluorescence quenching was also investigated and 3 mL for both drugs were determined to be optimum (Fig. 3B).

#### Effect of Eosin Y volume

**Fig. 4:**
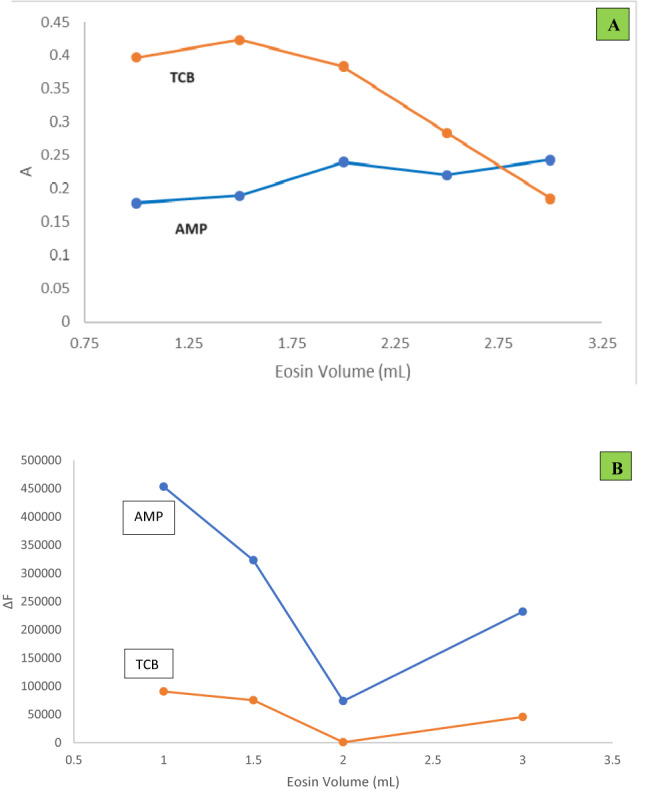
(**A**) Effect of Eosin Y volume on the absorbance readings (method Ι) of the formed complexes (3.0 µg/mL AMP and 5.0 µg/mL TCB) using the optimum experimental conditions. (**B**): Effect of Eosin Y volume on the ΔF values (method ΙΙ) of the formed complexes (1.0 µg/mL AMP and 0.5 µg/mL TCB) using the optimum experimental conditions.

Several volumes of Eosin Y solution (5 × 10^–3^ M) were tried to choose the optimum value. Regarding method I, it was determined that 2.7 mL and 1.5 mL of (5 × 10^–3^ M) Eosin Y was appropriate for AMP and TCB, respectively resulting in maximum absorbance values for the formed complexes (Fig. 4A). While for method II, it was determined that 1 mL of (5 × 10^–3^ M) Eosin Y was appropriate for either drugs resulted in maximum ΔF values (Fig. 4B).

#### Effect of reaction temperature

**Fig. 5:**
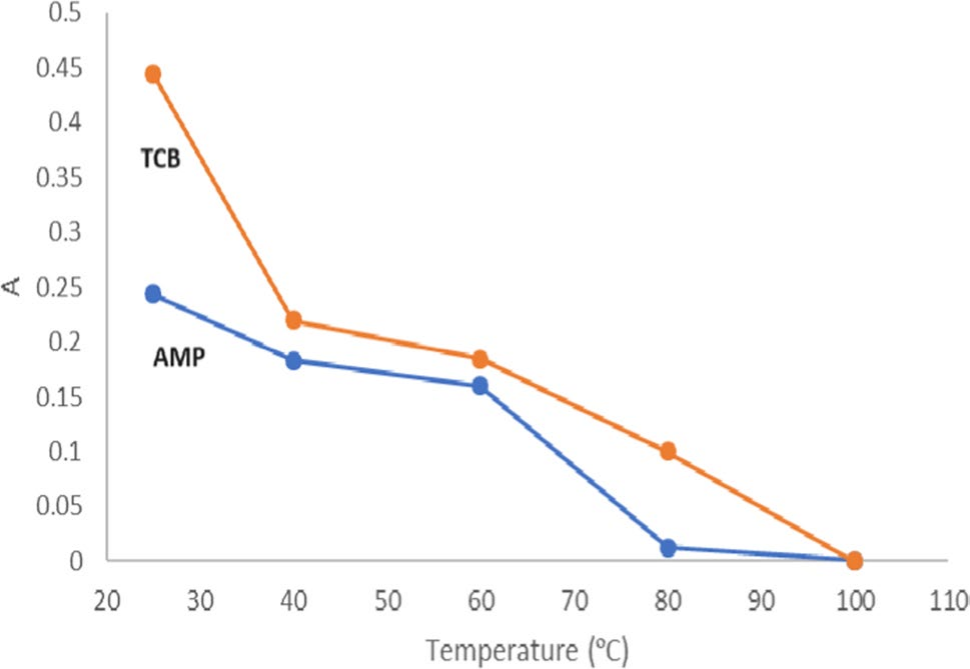
Effect of different temperature settings on the absorbance readings of the formed complexes (3.0 µg/mL AMP and 5.0 µg/mL TCB) applying optimum reactions conditions.

Regarding method I, the effect of different temperature settings on the complex’s absorbance was studied within the 25–100 ºC range. The absorbance measurements steadily drop when the reaction temperature was raised, with the maximum responses obtained at room temperature (Fig. 5). This effect might be explained by assuming that higher temperature settings negatively affect the stability of the formed complexes.

#### Effect of order of addition

Regarding method I, studying the effect of addition order on the absorbance of the produced complexes was carried out. It was discovered that adding Eosin Y or buffer solution first had significant effect on the absorbance readings. The highest absorbance values were achieved for both drugs when Eosin Y was added initially, hence this was followed through all steps of the designed assay. While for method II, the highest ΔF values were achieved for AMP when Eosin Y was added initially, but for TCB when buffer was added first, better results were obtained, hence this was followed through all steps of the proposed method.

#### Effect of reaction time

For method I, by measuring the absorbance of the reaction mixture every ten minutes, it was possible to investigate the effect of time on the complex’s formation. It was discovered that the complex is formed instantaneously and remains stable for two hours. while for method II, 15 min for both drugs were required for the reaction to be complete, hence this was followed during analysis.

### Reaction stoichiometry

**Fig. 6:**
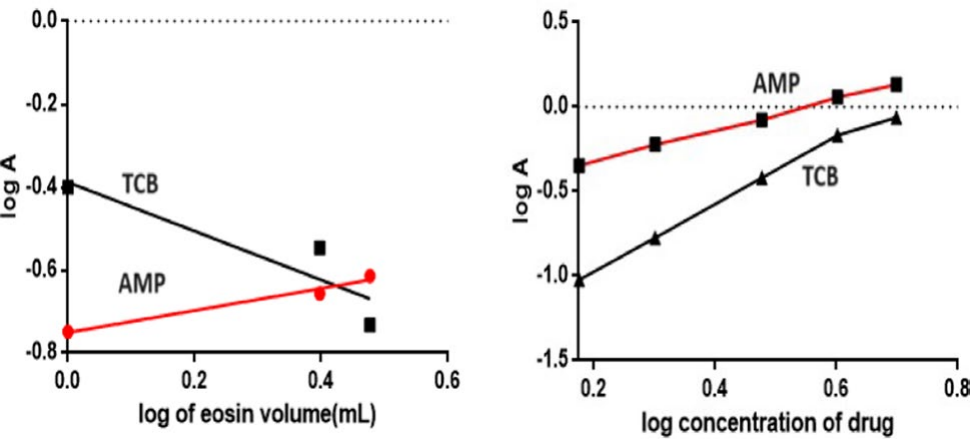
Reaction stoichiometry between AMP/TCB and Eosin Y (5.0 × 10^–3^ M) using limiting logarithmic method.

**Fig. 7:**
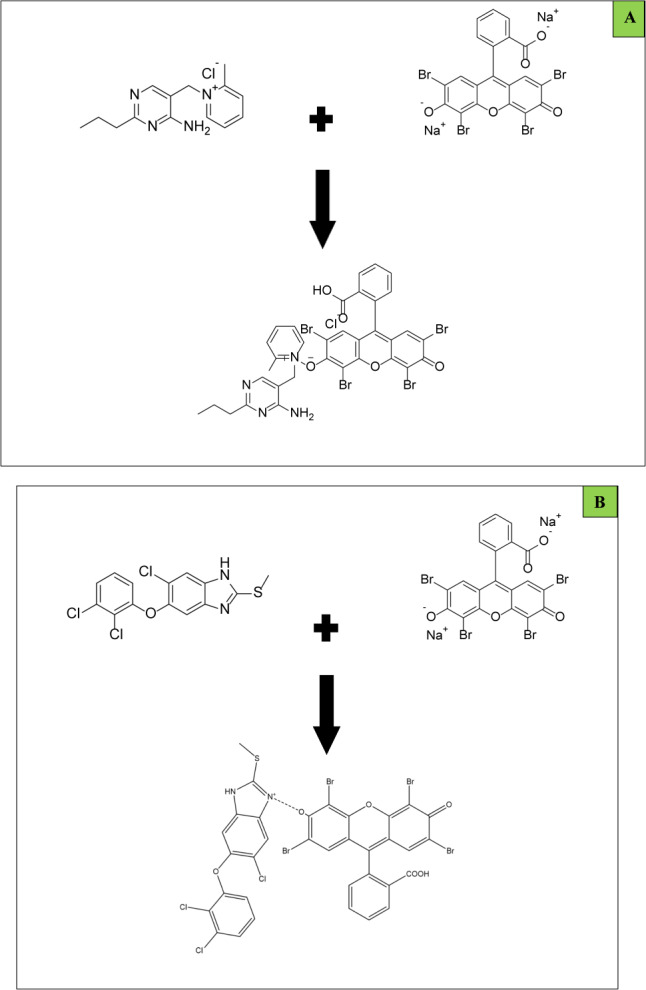
Proposal for the reaction mechanism between AMP (**A**) and TCB (**B**) with Eosin Y.

**Fig. 8:**
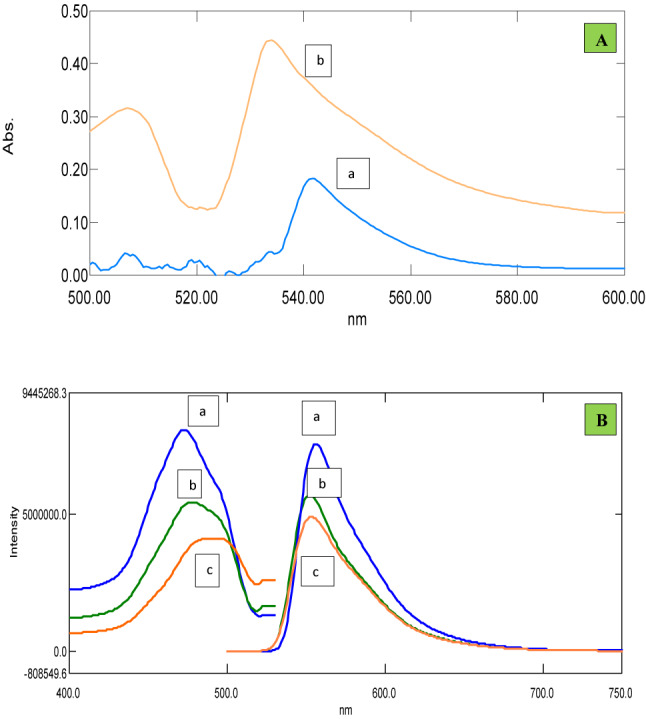
(**A**) Absorption spectrum of the reaction with Eosin Y using the optimum experimental conditions of: (**a**) Amprolium (3.0 µg/mL) in deionized water. (**b**) Triclabendazole (5.0 µg/mL) in methanolic solution. (**B**) Fluoroscence spectrum of the reaction with Eosin Y using the optimum experimental conditions of: (a, aˋ) excitation and emission spectra of Eosin Y (5 × 10^–3^ M) in acetate buffer. (b, bˋ) excitation and emission spectra of TCB (1.3 µg/mL) after reaction with Eosin Y using the optimum experimental conditions. (c, cˋ) excitation and emission spectra of AMP (0.5 µg/mL) after reaction with Eosin Y using the optimum experimental conditions.

The limiting logarithmic method^46^ was utilized to investigate the reaction stoichiometry between AMP and TCB with Eosin Y. graphing log absorbance readings versus either log [eosin Y] or log [AMP] yielded straight lines with slopes of 0.29/0.92. On the other hand, graphing log absorbance readings versus either log [eosin Y] or log [TCB] yielded slopes of 0.63/1.89 (Fig. 6). According to a literature review^47^, AMP and TCB have a tertiary amine group, which allows them to interact with negatively charged dyes and reagents. At an acidic pH, the tertiary nitrogen readily undergoes protonation, forming a positive center that engages in electrostatic interactions with the negative groups in dyes and reagents. The pKa1 and pKa2 values of Eosin Y, a weak acid, are 2.6 and 3.6, respectively^47^. It ionizes in distilled water to form a negative center in the molecule in an acidic medium. Consequently, the aliphatic chain’s nitrogen is readily protonated under certain acidic conditions, supplying one positive center. Thus, by hydrophobic forces and electrostatic attraction, Eosin Y: AMP + and TCB + will form a 1:1 neutral ion association complex. (Fig. 7A,B) illustrates the suggested course of the reaction pathway. After applying the optimum experimental conditions, absorption and fluorescence spectra of the ion pair complexes analytes applying both methods could be illustrated in (Fig. 8).

### Validation of the developed method

Under the guidance of ICH^48^, validation criteria such as linearity, range, and precision and robustness, as well as detection and quantitation limits; LOD and LOQ, were established.

#### Linearity and range

By plotting the absorbance (method I) or ΔF (method II) versus the final concentrations (ng/mL) produced calibration plots that were determined to be linear over the concentration ranges illustrated in (Table 1). The statistical regression line stated the validity of the proposed methods, and it was showed that the calibration plots had good linearity indicated by the high correlation coefficient values (r^2^) and small percentage relative standard deviation (% SD) (Table 2).

**Table 2:** Analytical performance data for the determination of Amprolium and Triclabendazole applying the proposed methods.

Instrument				
Parameters	Spectrophotometry (Method Ι)		Spectrofluorimetry (Method ΙΙ)	
	AMP	TCB	AMP	TCB
Concentration range (ng/mL)	100–5000	1500–10,000	500–2000	100–1500
Limit of detection LOD (ng/mL)	45	191	142	67
Limit of Quantitation LOQ (ng/mL)	136	579	429	202
Regression equation	Y = 0.2818x + 0.0189	Y = 0.2952x- 0.4121	Y = 281969x + 274,352	Y = 309213x + 4346.7
Correlation coefficient	0.9999	0.9997	0.9955	0.9984
S.D. of the residuals (Sy/x)	5.5 × 10^-3^	1.89 × 10^-2^	11.72 × 10^-3^	7.94 × 10^-3^
S.D. of intercept (Sa)	3.8 × 10^-3^	1.7 × 10^-2^	12.1 × 10^-3^	6.23 × 10^-3^
S.D. of the slope (Sb)	1.9 × 10^-3^	2.7 × 10^-3^	9.5 × 10^-3^	7.1 × 10^-3^

#### LOQ and LOD

The lowest drug concentration that can be identified under the stated experimental conditions but not necessarily quantified is known as the limit of detection (LOD). The lowest analyte concentration that can be identified with acceptable accuracy and precision is known as the limit of quantification (LOQ). The limits of quantification (LOQ) and limits of detection (LOD) were determined according to ICH Q2 (R1) recommendations^48^. The calculated values are listed in (Table 2).

LOQ and LOD were calculated according to the following equations:

$${\text{LOQ }} = { 1}0{\text{ S}}_{{{\text{a}}/{\text{b}}}}$$

$${\text{LOD }} = { 3}.{\text{3 S}}_{{{\text{a}}/{\text{b}}}}$$

where S_a_ is the standard deviation of the intercept of regression line and b is the slope of the regression line.

#### Precision

Three replicate analyses of three concentrations of pure AMP and TCB were used to assess the intra-day precision. Through replicate analyses of three concentrations over three consecutive days, the inter-day precision was also assessed (Table 3) provides a summary of the intra-day and inter-day precision results. The suggested method’s high precision is indicated by the small SD values.

**Table 3:** Precision data for the determination of Amprolium and Triclabendazole applying the proposed methods.

Spectrophotometry (Method Ι)							
Intra-day precision				Inter-day precision			
AMP		TCB		AMP		TCB	
Conc. taken (ng/mL)	Mean* %found ± SD	Conc. taken (ng/mL)	Mean* %found ± SD	Conc. taken (ng/mL)	Mean* %found ± SD	Conc. Taken (ng/mL)	Mean* %found ± SD
100	100.78 ± 0.66	1500	102.32 ± 0.22	100	99.79 ± 0.1	1500	102.22 ± 0.29
2500	99.51 ± 1.42	6000	100.42 ± 0.86	2500	99.57 ± 0.11	6000	100.77 ± 0.15
5000	99.43 ± 0.71	10,000	99.32 ± 0.34	5000	99.54 ± 0.14	10,000	99.33 ± 0.33

Spectrofluorimetry (Method ΙΙ)							
Intra-day precision				Inter-day precision			
AMP		TCB		AMP		TCB	
Conc. Taken (ng/mL)	Mean* %found ± SD	Conc. Taken (ng/mL)	Mean* %found ± SD	Conc. Taken (ng/mL)	Mean* %found ± SD	Conc. Taken (ng/mL)	Mean* %found ± SD
500	100.09 ± 1.16	100	99.61 ± 0.47	500	98.87 ± 0.82	100	101.05 ± 0.23
1000	101.07 ± 1.07	1000	100.76 ± 1.11	1000	99.69 ± 0.67	1000	99.92 ± 0.34
2000	98.20 ± 0.41	1500	98.64 ± 0.31	2000	99.23 ± 0.38	1500	99.25 ± 0.87

#### Robustness

Consistency of absorbance (method I) or ΔF (method II) with deliberately minor changes in various experimental conditions was used to establish it. These changes related to methods I and II and involve the buffer pH and the Eosin volume as shown in (Figs. 2A and 4A). The robustness of these methods was demonstrated by the fact that neither the ΔF nor the absorbance of the drugs under study were impacted by these little changes that may happen during the experimental process.

### Applications

The developed method was successfully applied to assay AMP and TCB in pharmaceutical wastewater applying both methods. The outcomes demonstrated that the developed method show satisfactory accuracy as revealed from the high percent recoveries (Table 4).

**Table 4:** Assay results for the determination of Amprolium and Triclabendazole in Pharmaceutical waste water applying the proposed methods.

Spectrophotometry (Method Ι)						
Parameters	Amprolium			Triclabendazole		
Waste water	Conc. taken (ng/mL)	Found	% Recovery	Conc. taken (ng/mL)	Found	% Recovery
	700	684	97.71	2000	1995	99.75
	1000	1020	102	3000	2948	98.27
	1500	1500	100	6000	6114	101.9
	2500	2466	98.64	8000	8141	101.76
	3000	3022	100.73	10,000	9861	98.61
Mean ± SD			99.82 ± 1.69			100.06 ± 1.71

Spectrofluorimetry (Method ΙΙ)						
Parameters	Amprolium			Triclabendazole		
Waste water	Conc. taken (ng/mL)	Found	% Recovery	Conc. taken (ng/mL)	Found	% Recovery
	500	490	98	300	293	97.6
	600	590	98.3	500	510	102
	700	710	101.4	700	690	98.5
	1300	1330	102.3	1000	1010	101
	1500	1460	97.3	1300	1280	98.46
Mean ± SD			99.46 ± 2.23			99.51 ± 1.88

## Conclusion

Using a straightforward and accurate visible spectrophotometric and spectrofluorimetric techniques based on a water-soluble ion-pairing complex with Eosin Y, AMP and TCB were identified and quantified in pharmaceutical waste water. The suggested methods have several benefits, including simplicity, time savings, lack of complicated treatments or laborious extraction techniques, and comprehensive validation, making it suitable for routine analysis in quality control laboratories.

## Data Availability

The datasets generated and/or analyzed during the current study are available from the corresponding author upon reasonable request.
